# Non-patchy strategy for inter-atomic distances from Extended X-ray Absorption Fine Structure

**DOI:** 10.1038/srep42143

**Published:** 2017-02-09

**Authors:** Gu Xu, Guifang Li, Xianya LI, Yi Liang, Zhechuan Feng

**Affiliations:** 1 Department of Materials Science and Engineering, McMaster University, Hamilton, Ontario, L8S4L7, Canada; 2 School of Advanced Materials and Nanotechnology, Xidian University, Xi’an, Shaanxi, China; 3 Laboratory of optoelectronic materials & detection technology, Guangxi Key Laboratory for Relativistic Astrophysics, School of Physics Science and Technology, Guangxi University, Nanning, China

## Abstract

Extended X-ray Absorption Fine Structure (EXAFS) has been one of the few structural probes available for crystalline, non-crystalline and even highly disordered specimens. However, the data analysis involves a patchy and tinkering process, including back-and-forth fitting and filtering, leading to ambiguous answers sometimes. Here we try to resolve this long standing problem, to extract the inter-atomic distances from the experimental data by a single step minimization, in order to replace the tedious and tinkering process. The new strategy is built firmly by the mathematical logic, and made straightforward and undeniable. The finding demonstrates that it is possible to break off from the traditional patchy model fitting, and to remove the logical confusion of a priori prediction of the structure to be matched with experimental data, making it a much more powerful technique than the existing methods. The new method is expected to benefit EXAFS users covering all disciplines. Also, it is anticipated that the current work to be the motivation and inspiration to the further efforts.

As a unique method of probing short range order in atomic scale, Extended X-ray Absorption Fine Structure (EXAFS) is routinely used in a wide range of scientific fields, including biology, environmental science, catalysts research, and material science^1^. It has even made profound impact on our understanding of the local metrical structure of the active sites in many metallo-proteins^2^, as well as been utilized extensively to provide unique structural insights into enzymatic intermediates^3^. More importantly, crystallinity is not required for EXAFS measurements, making it one of the few structural probes available for non-crystalline and highly disordered materials, including solutions^1^,^4^. As an atomic probe, it places few constraints on the samples that can be studied. Since all atoms have core level electrons, EXAFS spectra can be measured for almost every element on the periodic table^4^.

However, as a long standing problem, in order to extract the results from the EXAFS data, such as inter-atomic distances, the current state-of-art calls for a patchy and tinkering process, when one has to go back-and-forth to perform the fitting and filtering^5^,^6^,^7^,^8^, including the Fourier transform^9^,^10^ of individual coordination shell, leading to a possibly wrong answer due to the interference, whereas the simultaneous fitting of multiple coordination shells involves too many variables and becomes unreliable^11^,^12^,^13^,^14^.

To resolve the problem, we develop here a non-patchy, non-ambiguous strategy of obtaining the inter-atomic distances, which no longer relies on the Fourier transform. Our new method is based on the mathematical analysis of existing theory, the cause of the problem in the current state-of-art, in particular the discrepancy between the individual coordination shell and the overall Fourier transform, which is then reverted to become the key to the development of new strategy, where a single calculation step generates the needed results without ambiguity. In addition to the verification by the existing data, the new strategy brings in extra surprises where some variables may be ignored initially, making it a much more powerful tool than the existing methods.

As usual the EXAFS data are collected from synchrotron x-ray facilities either in transmission or fluorescence geometries^1^,^4^,^6^,^7^. The X-ray absorption coefficient μ(E), expressed as a function of energy, E, is normally converted into χ(k), to highlight the fine structure, i.e., the oscillating part of the X-ray absorption, where k is the wave number of the photo-electrons, given by (2 m(E-E_0_)/ħ^2^)^1/2^, and E_0_ marks the absorption edge^1^,^4^ (Fig. 1).

According to the standard theory^6^,^7^,^8^,^9^,^10^, the χ(k) is related to a number of terms, such as; S_0_, the amplitude reduction due to the relaxation of all the other electrons in the absorbing atom to the hole in the core level; f_j_(k) and φ_j_(k), the scattering amplitude and phase-shift of the atoms in the j_th_ coordination shell neighboring the excited atom; λ(k), the mean-free-path of the photo-electron; N_j_, the number of neighboring atoms; R_j_, the distance from the absorbing to the neighboring atoms; and σ_j_^2^, the disorder in the neighboring atoms of the j_th_ coordination shell; as shown by Eqn (1):


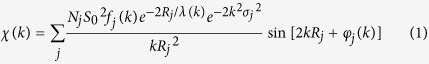


To achieve the main goal of obtaining the structural information, it is possible, at least in principle, to extract the desired results by the data fitting, the inter-atomic distances R_1_, R_2_, (or more precisely the respective scattering path lengths), the coordination numbers N_1_, N_2_, and the disorder factor σ_j_, based on the phase information φ_j_(k), scattering amplitude f_j_(k) and photo-electron’s mean-free-path, λ(k), which could all be calculated beforehand^1^,^4^,^7^. However, because there are too many unknowns here, it is usually very difficult, if not impossible, to achieve the correct results by this data fitting, unless much more has already been known a priori, thus beating the original purpose. As a result, the current state-of-art calls for the separation of coordination shells through Fourier transform, to “isolate” the individual peaks of the χ(R), the Fourier transform of χ(k), to be linked to each coordination shell surrounding the absorbing atom. The overall process becomes inevitably tinkering and patchy, when one has to go back-and-forth to perform the fitting and filtering^14^,^15^,^16^,^17^,^18^,^19^,^20^,^21^. Although there have been non-Fourier methods attempted, but none solves the problem, or there would have been no need for the Fourier Transform till this day.

Moreover, this is mathematically problematic, since a number of single peaks added together may not necessarily produce a multiple peak which matches the peak positions of the original, due obviously to the possible interference and complications caused by the peak shoulders. For example, when we Fourier transform the two sine functions, simulating the oscillating part of χ(k) using a typical set of phases (Fig. 2a), it is evident that the sum of the two does not match each individual transform in terms of the peak positions (Fig. 2b). The mismatch arises due to the existence of peak shoulders, viz., the interference between the truncated coordination shells. In addition, there may also be extra peaks emerging from the summation caused by the truncation error, such as the one found in between the two major peaks (Fig. 2b). Therefore, it becomes clear that, the Fourier transform of individual coordination shells may lead to possibly wrong answers. Worse off, one could never tell beforehand, when this happens, how much different it would be, or even the direction of the deviation, all of which place the whole process onto a logically fallacious position.

To resolve the problem, let us first break off from the traditional route, by redirect the attention onto the zeros of the χ(k), as they are the key features – oscillations of EXAFS, and in fact the key to the solution. When we consider a zero-point of the χ(k_m_), where k_m_ (m = 1,2,3.) are the roots of χ(k), it is obvious that, the multiples to the right side of Eqn (1) become unimportant, as a zero times anything is still zero. For example, when there are only two coordination shells involved, Eqn (1) can just be re-written as the sum of merely two sine functions:


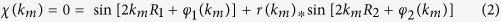


where r(k) is:


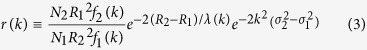


Obviously Eqn (2) is much simplified from Eqn (1), and the tedious multiple parameter fitting is now reduced to a set of algebraic equations, of just four unknowns of, R_1_, R_2_, N_2_/N_1_, and (σ_2_^2^–σ_1_^2^). Indeed, with a given set of φ_j_(k_m_), f_j_(k_m_) and λ(k_m_), it is possible to employ Eqn (2) four times, each with a different zero point of k_m_, to solve for the unknowns from the equation set.

Rather than solving these transcendental equations, however, a much better strategy can be developed here, to obtain atomic distances of R_1_ and R_2_ directly, by a minimization algorithm. Due to the oscillating nature of sine functions, for a given root of k_m_, Eqn (2) produces a number of zeros, or minima when the square of χ(k_m_) is constructed, on a two dimensional mesh spanned by R_1_ and R_2_ (Fig. 3a). They form the periodical solutions of Eqn (2), where the “roots” of R_1_ and R_2_ are separated by a “period” of π/k_m_ along R_j_ axes. The periodicity is not changed by the presence of the phases φ(k) included in the sine function, which would only make it less harmonic, or broaden the peak spectra, when taken as a sinusoidal function of k instead of R.

However, when a number of such squared χ(k_m_) are added together, each corresponding to a different periodicity π/k_m_, the resulting sum becomes aperiodic. In particular, when up to 5 or more such χ^2^(k_m_) are collected, the summation produces few minima instead of periodic, often with the smallest corresponding to the correct solution of R_1_ and R_2_ (Fig. 3b,c), as long as physically meaningful boundaries of R_j_ are imposed. Naturally, this can also be expanded to include a third or even fourth coordination shell, when Eqn (2) involves more sine functions of 2kR_3_, 2kR_4_, etc.

In the meantime, a prominent feature of the new method can be discovered here; these minima are so insensitive to, but not totally independent of, the change of r(k), as given by Eqn (3), that the minimization result varies little with respect to the choice of r(k), which consists of non-zero factors anyway. In fact, it is easy to verify that, except for the relative heights, the locations of the minima are almost unchanged by a variation of r(k) up to 25%, which is about the maximum change of r(k_m_), caused by k_m,_ which varies from 1.5–5.0, and the accompanying f_j_(k_m_), λ(k_m_). This implies, we do not need to know, a priori, the knowledge of f_j_(k) and λ(k), although they can be calculated beforehand. This pleasant surprise can be understood by the fact that, R_j_ is only related to φ_j_(k), through the sine function in the theory, which decides where the zeros are, and has little to do with the scattering amplitude and the mean-free-path. Nonetheless, the ratio(s), r(k), could well be set as extra variables, regardless the number of shells, and/or k ranges. Of course, we are able to figure out the N_j_, as well as σ_j_, through the usual fitting process afterwards, which becomes much less ambiguous now, due to the settlement of R_1_, R_2_. In any case, the new method provides a much more powerful tool to achieve the most needed answer, the atomic distances, without ambiguity. And with these answers, the tedious, patchy job can largely be reduced, to allow for the further refinement.

Based on the logical analysis above, the experimental/numerical verification becomes trivial. The new strategy can be tested by a common software, such as Matlab™, where there are a number of simple, built-in loops, such as “**f**minsearch” based on Nelder-Mead minimization algorithm^22^. It takes only one line of instruction in Matlab™ command window. As an example, we employ the existing data of FeO (Fig. 4a), involving non-monotonic phase functions due to the Ramsauer-Townsend effect, which produced a number of zeros of χ(k) at; k_m_ = 1.80, 2.10, 2.40, 3.25, 4.10, 4.90, (m = 1..6)^23^,^24^. We then construct the target to be minimized by adding up the squares of the right hand side of Eqn (2), for each k_m_, together with the given φ(k_m_)^23^,^24^, and a common r for all m = 1..6. It takes only a few seconds for the Matlab™ on a Pentium to generate the correct answers of R_1_ = 2.14 Å, and R_2_ = 3.06 Å, respectively, ending with a residual of about exp(−4.5), or 0.011 (Fig. 3c). The same procedure is then repeated to allow for the variations of all 6 r(k_m_), when a much lower residual of 6 × 10^−10^ can be achieved. And the corresponding r(k_m_) are shown in Fig. 4b.

To obtain convincing evidence for the legitimacy of the procedure, Fig. 5a and b are presented to show a), the variation of χ^2^(k_1_) against R_1_, involving only the first root (k_1_) of χ, and b), the variation of Σχ^2^(k_j_), the sum over all roots (k_m_) of χ, both with R_2_ near the correct answer of 3.06 Å. It is clear that the former gives roughly close results but still with ambiguity, whereas the latter produces a unique and un-mistakable answer.

The same can be done to compare the variation of χ^2^(k_1_) against R_2_, by Fig. 6a, with that of Σχ^2^(k_j_), by Fig. 6b, when only a common r is used in the minimization, and with that by Fig. 6c, when all 6 r(k_m_) are employed, all with R_1_ near the correct answer of 2.14 Å. Although a single minimum is visible in Fig. 6a, it does not represent the correct answer, which will be better obtained by Fig. 6c, where all the possible variations are involved.

Since the error is mainly originated from the data input, as the minimization process does not introduce additional uncertainty, the overall % error remains the same as the k_m_. Although the k_m_ accuracy affects the analysis, this influence is much less than the old method, when the whole oscillation curve is employed. Of course, as with many minimization routines, we have to establish sensible initial values of R_1_, R_2_, and to avoid being trapped into local minima. An easy choice could be to require R_2_ > R_1_ during the searching, and/or to preset physically meaningful R-values to begin with, e.g., letting R_1_ to be within {1,3}, and R_2_ within {2,4} (Fig. 3b). The new method has also been tested by a number of other samples and the results were consistent, which confirm the correct mathematics. Even when more R’s than the actual inter-atomic distances are employed in the procedure, the correct results can still be achieved, where the extra R’s will be associated with negligible r’s after the minimization. As an example, a triple shell case of AlGaN was also tested, where k_m_ = 2.47, 3.53, 4.51, 5.85, 6.23, 6.96, 8.15, 9.29, the target to be minimized was then constructed by the same manner, and the resulting R_1_, R_2_, R_3_ match well the correct answers: 1.91, 3.11, 3.14, respectively. Mathematically, as long as the theory is true (Eqn.1 & 2), correct answers should be expected, unless there is an input error. Nevertheless, a further test was done to the system of SiC, involving a certain degree of disorder. With the following roots of χ: 1.07, 1.85, 2.65, 3.54, 4.35, 4.68, 5.23, 5.93, the minimization process produces a pair of R_1_/R_2_ to be 1.83/3.03, both a little smaller than the crystalline results (1.90/3.06).

To summarize, a simple and straightforward strategy has been proposed here to extract the main structural information, such as the inter-atomic distances, from the EXAFS, which has otherwise required a lengthy tinkering and patchy process. Our findings demonstrate that it is now possible to break off from the various patching of the Fourier transform method, and to remove the mathematical flaw of separating coordination shells to be matched with experimental data, which has been dominating for many years. Even though one may still need to employ curve-fitting for other parameters, however, the new method still offers tremendous benefit, since, once the inter-atomic distances are settled, the problem becomes solvable, whereas it may be impossible by the curve-fitting alone. We hope the new method will re-establish the EXAFS as a far less ambiguous technique during the data analysis. We believe that the new method has far reaching implications for the use of EXAFS technique, not only in physical sciences but also in life sciences, where EXAFS analysis plays an important role.

## Additional Information

**How to cite this article**: Xu, G. Non-patchy strategy for inter-atomic distances from Extended X-ray Absorption Fine Structure. *Sci. Rep.* **7**, 42143; doi: 10.1038/srep42143 (2017).

**Publisher's note:** Springer Nature remains neutral with regard to jurisdictional claims in published maps and institutional affiliations.

## Figures and Tables

**Figure 1:**
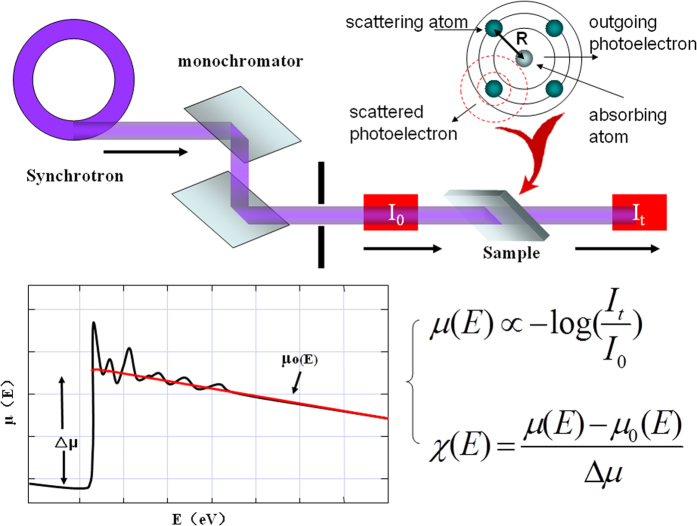
EXAFS experimental setup for the transmission mode, the absorption energy oscillation, and the data extraction, where the jump in μ(E) marks the absorption edge.

**Figure 2:**
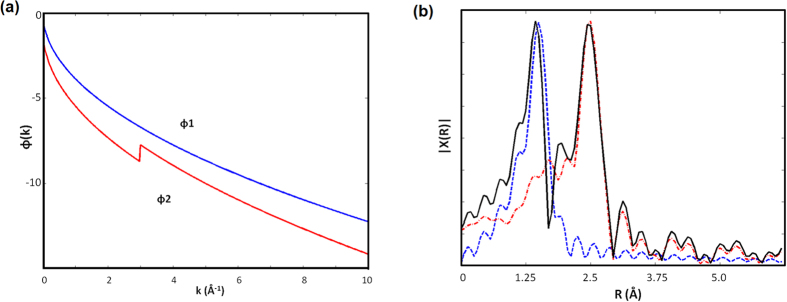
(**a**) Examples of the scattering phases between an absorbing atom and scattering atoms. (**b**), the mismatch between the sum of the two Fourier transforms (black), and the individual transform (blue: dash; red: dash-point), where the peak positions differ due to the peak shoulders.

**Figure 3:**
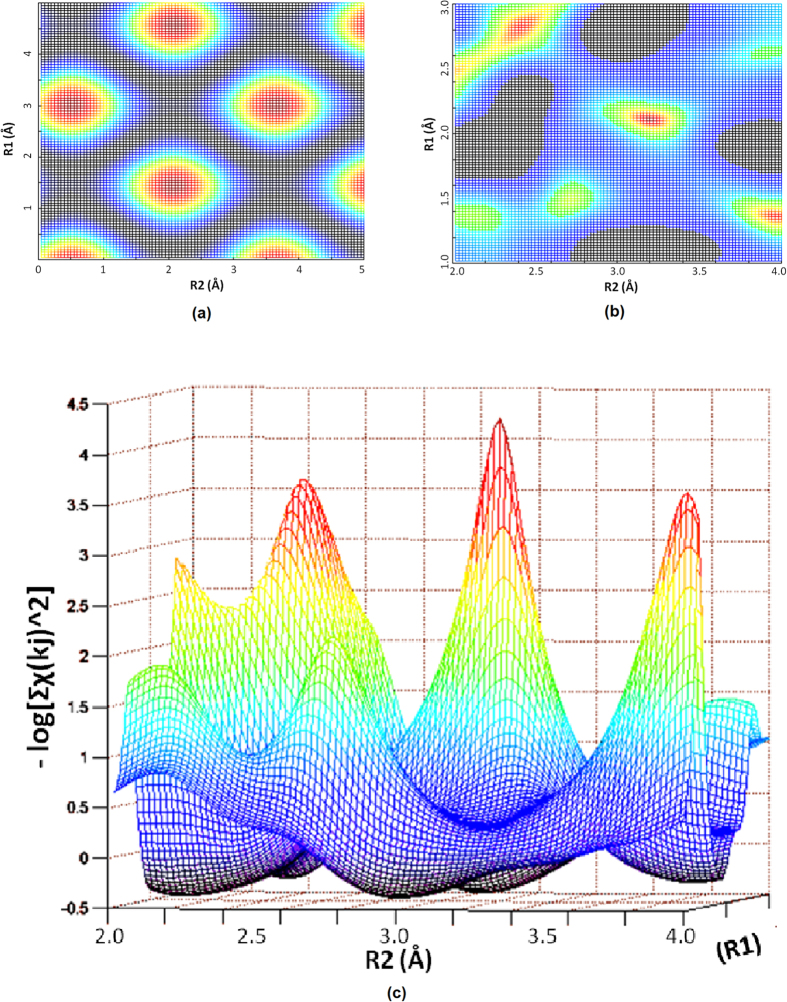
(**a**) A periodical set of minima (dark-red) on a two dimensional mesh spanned by R_1_ and R_2_, caused by the oscillating nature of sine functions in Eqn (2) when the square of χ(k_m_) is constructed by k_m_ = 1.0. (**b**) The summation of χ^2^(k_j_) over five k_m_’s, instead of periodic, it produces the smallest minimum (dark red) corresponding to the solution of R_1_ and R_2_. (**c**) Three dimensional view of (**b**), where the smallest minimum is shown by the highest peak in red-black.

**Figure 4:**
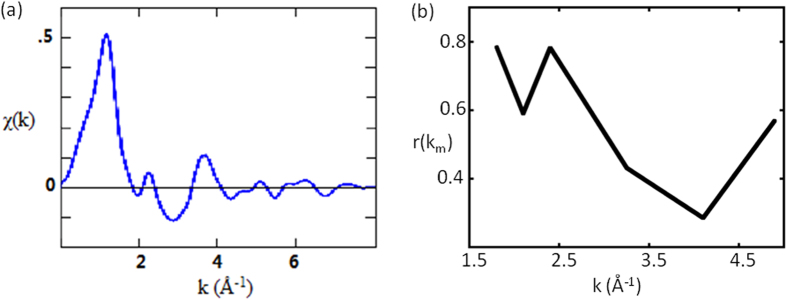
(**a**) χ(k) from the EXAFS data of FeO. (**b**) r(k_m_) obtained from the proposed minimization process.

**Figure 5:**
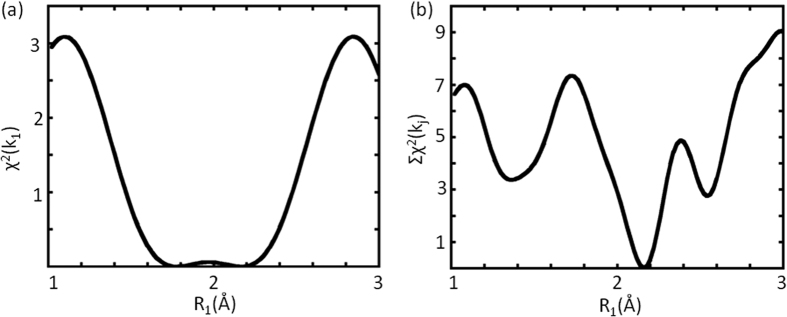
(**a**) The variation of χ^2^(k_1_) against R_1_, involving only the first root (k_1_) of χ, with R_2_ near the correct answer, giving close results but still with ambiguity. (**b**) The variation of Σχ^2^(k_j_), the sum over all roots (k_m_) of χ, with R_2_ near the correct answer, producing a unique and un-mistakable answer.

**Figure 6:**
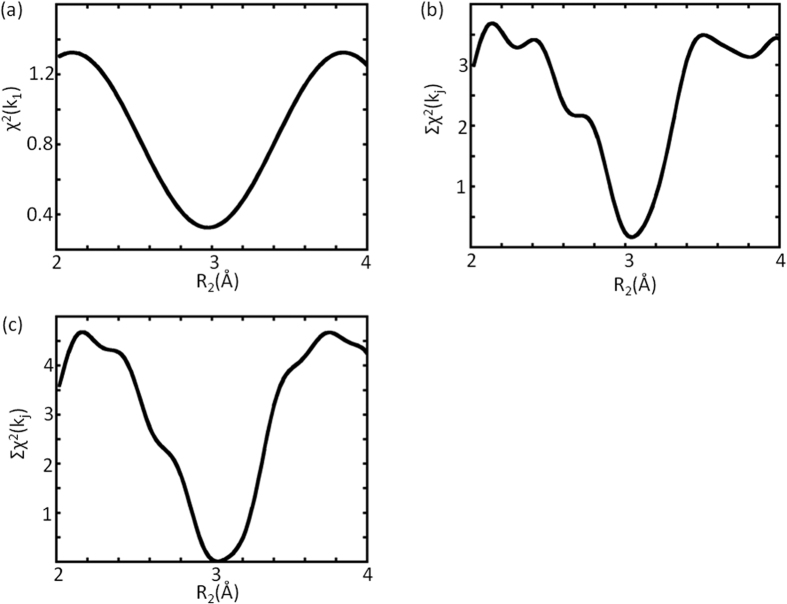
(**a**) The variation of χ^2^(k_1_) against R_2_, involving only the first root (k_1_) of χ, with R_1_ near the correct answer, giving close results but a little off the correct answer. (**b**) The variation of Σχ^2^(k_j_), the sum over all roots (k_m_) of χ, with R_2_ near the correct answer, but using only a common r during the minimization. (**c**) The variation of Σχ^2^(k_j_), the sum over all roots (k_m_) of χ, with R_2_ near the correct answer, employing all r(k_m_) as variables.
